# Chemical characterization, cytotoxic, antioxidant, antimicrobial, and enzyme inhibitory effects of different extracts from one sage (*Salvia ceratophylla* L.) from Turkey: open a new window on industrial purposes

**DOI:** 10.1039/d0ra10044g

**Published:** 2021-01-28

**Authors:** Sengul Uysal, Gokhan Zengin, Kouadio Ibrahime Sinan, Gunes Ak, Ramazan Ceylan, Mohamad Fawzi Mahomoodally, Ahmet Uysal, Nabeelah Bibi Sadeer, József Jekő, Zoltán Cziáky, Maria João Rodrigues, Evren Yıldıztugay, Fevzi Elbasan, Luisa Custodio

**Affiliations:** Erciyes University Halil Bayraktar Health Services Vocational College Kayseri Turkey senguluysal@erciyes.edu.tr; Drug Application and Research Center, Erciyes University Kayseri Turkey; Physiology and Biochemistry Research Laboratory, Department of Biology, Science Faculty, Selcuk University Campus Konya Turkey; Department of Health Sciences, Faculty of Medicine and Health Sciences, University of Mauritius Réduit Mauritius; Department of Medicinal Laboratory, Vocational School of Health Services, Selcuk University Konya Turkey; Agricultural and Molecular Research and Service Institute, University of Nyíregyháza Nyíregyháza Hungary; Centre of Marine Sciences, University of Algarve, Faculty of Sciences and Technology Ed. 7, Campus of Gambelas 8005-139 Faro Portugal; Department of Biotechnology, Science Faculty, Selcuk University Campus Konya Turkey

## Abstract

In the present study, the methanolic, hydro-methanolic, dichloromethane, hexane and aqueous extracts of *Salvia ceratophylla* L. (Family: Lamiaceae), a lemon-scented herb, were tested for total phenolic (TPC) and flavonoid content (TFC) and antioxidant activities were evaluated using a battery of assays (2,2-diphenyl-1-picrylhydrazyl (DPPH), 2,2-azino-bis(3-ethylbenzothiazoline-6-sulfonic acid) (ABTS), ferric reducing antioxidant power (FRAP), cupric reducing antioxidant capacity, total antioxidant capacity (TAC) (phosphomolybdenum) and metal chelating). Enzyme inhibitory effects were investigated using acetyl- (AChE), butyryl-cholinesterase (BChE), tyrosinase, α-amylase and α-glucosidase as target enzymes. Regarding the cytotoxic abilities, HepG2, B164A5 and S17 cell lines were used. The phytochemical profile was conducted using liquid chromatography-mass spectrometry/mass spectrometry (LC-MS/MS). Our data showed that the methanolic aerial extracts possessed the highest phenolic (72.50 ± 0.63 mg gallic acid equivalent per g) and flavonoid (43.77 ± 1.09 mg rutin equivalent per g) contents. The hydro-methanolic aerial extract showed significant DPPH radical scavenging activity (193.40 ± 0.27 mg TE per g) and the highest reducing potential against CUPRAC (377.93 ± 2.38 mg TE per g). The best tyrosinase activity was observed with dichloromethane root extract (125.45 ± 1.41 mg kojic acid equivalent per g). Among the tested extracts, hexane root extract exerted the highest antimicrobial potential with a minimum inhibitory concentration value of 0.048 mg mL^−1^. Methanolic root extract showed the lowest cytotoxicity (28%) against HepG2 cells. Phytochemical analysis revealed the presence of important polyphenolic compounds including luteolin, gallic acid, rosmarinic acid, to name a few. This research can be used as one methodological starting point for further investigations on this lemon-scented herb.

## Abstract 2

Our findings suggested that *Salvia ceratophylla* could be one potential raw material in industrial applications.

## Graphical abstract

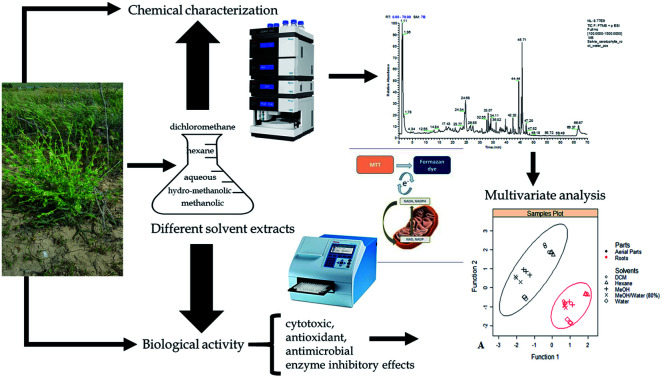

## 1. Introduction

*Salvia ceratophylla* L. (*S. ceratophylla*) is a biennial lemon-scented herb belonging to one of the largest genera of the Lamiaceae comprising of about 900 species distributed worldwide.^1^ The herb is native to numerous places such as Afghanistan, Iran, Iraq, Lebanon-Syria, Palestine, the Transcaucasus, Turkey, and Turkmenistan.^2^ Published literature reported that a number of different *Salvia* species and their respective essential oils have showed promising pharmacological propensities namely antioxidant, cytotoxicity,^3^ antibacterial, anti-neurodegenerative,^4^ anti-enzymatic (anticholinesterase, anti-urease, anti-tyrosinase, anti-elastase),^5^ anti-tumour^6^ and antidiabetic activities^7^ to name a few. The herb, *S. ceratophylla*, in particular, is aromatic and in a recent analysis its essential oil (EO) was reported to possess anti-trypanosomal effects resulting with an inhibitory concentration (IC) 50 of 2.65 μg mL^−1^. The hexane extract demonstrated cytotoxicity activity against mouse erythroleukemia (MEL), KB (containing human papillomavirus 18 (HPV-18)), BT-549 (human breast cancer cell line), SK-OV-3 (human ovarian cancer cell line), LLC-PK1 (renal epithelial cell line) and VERO (kidney epithelial cell line) cell lines with IC50 values ranging from 60 to 100 μg mL^−1^. There are further data concerning antioxidant and chemical composition of the EO.^8–11^ The review of Ulubelen^12^ stated that the terpenoids present in *S. ceratophylla* exhibited interesting antibacterial activity. The work of Goren *et al.*^13^ also showed that the diterpenoids identified from the root of the herb exhibited strong antibacterial activity against *Staphylococcus epidermidi*s and *Proteus mirabilis*. Furthermore, two *seco*-4,5-abietane diterpenoids showed cytotoxic effects against MOLT-4 (human acute T lymphoblastic leukaemia cells) and MCF-7 (human breast cancer cell line) cell lines.^14^ In another study, the chloroform extract of *S. ceratophylla* significantly depressed anti-butyrylcholinesterase activity with a percentage inhibition of 91.3%.^15^ Based on ethnobotanical information, *S. ceratophylla* were used to treat cancers, infections, urinary complications,^8^ inflammation, and even nociceptive disorders.^8,16,17^ The World Health Organisation outlines that cancer is the second leading cause of death across the globe with 9.6 million deaths recorded in the year 2018.^2^ Despite cancer is one of the most studied disease and the clinical care and technology have advanced greatly, yet cancer remains still incurable.^18^ Natural products have been the only storehouse of pharmaceuticals for decades and have contributed enormously in human health through effective and unique bioactive compounds. Oxidative stress involving free radicals is the onset of several chronic diseases including cancers, neurological disorders, and cardiovascular diseases.^19^ Medicinal plants act as a major reserve of pharmaceuticals since the early days of mankind. Today, more than 80% of medicines are directly or indirectly linked to medicinal plants due to their strong pharmacological properties, low toxicity and low cost.^20^ On many occasions, natural enzyme inhibitors isolated from medicinal plants have been acknowledged as useful therapeutic tools for the management of numerous human pathologies.

Therefore, the quest for novel and efficient drugs from medicinal plants should be an ongoing process and a continuing need. For this reason, we evaluated the aerial part and root extracts of *S. ceratophylla* prepared from polar and non-polar solvents for their antioxidant, anti-enzymatic [acetylcholinesterase (AChE), butyrylcholinesterase (BChE), amylase, glucosidase, tyrosinase], anti-microbial and cytotoxicity activities. To the best of our knowledge, this is the first time the polar and non-polar extracts of this plant will be evaluated for the aforementioned studies and compiled in one single research work. The total phenolic and flavonoid content were quantified and the prepared extracts were screened for phytochemicals using liquid chromatography-mass spectrometry/mass spectrometry (LC-MS/MS) in order to correlate the observed biological activities with the biomolecules present. We believe that this study will add information on *S. ceratophylla* that can be used for further investigations.

## 2. Materials and methods

### 2.1. Plant material and preparation of extracts

*Salvia ceratophylla* samples were collected from natural population (Akyer village, Bozdağ national park, 1020 m, and steppes areas) in the summer period of 2019. Botanical identification was performed by one of the co-authors (Dr Evren Yıldıztugay) and a voucher specimen was kept at the herbarium of Selcuk University (EY-3005). Aerial parts and roots were carefully separated, dried in a shade for ten days, and then grinded by using a laboratory mill.

Different extracts were used in this study. To this end, powdered aerials parts and roots (5 g) were extracted in *n*-hexane, dichloromethane (DCM), methanol, methanol–water (80%) (100 mL) under stirring for 24 h at 25 °C. After that, the solvents were removed by a rotary evaporator and the extracts stored at 4 °C until analysis. Regarding aqueous extracts, we used traditional infusion technique and the plant material (5 g) were kept with 100 mL of boiled water. The extracts were filtered and then lyophilized. All extracts were stored at 4 °C in a refrigerator. The extraction yields (%) are given in Table 1.

**Table 1:** Extraction yields (%), total phenolic and flavonoid content of *Salvia ceratophylla* extracts^a^

Parts	Solvents	Yield (%)	TPC (mg GAE per g)	TFC (mg RE per g)
Aerial parts	Hexane	4.0	17.33 ± 0.10^h^	5.25 ± 0.12^f^
	DCM	4.96	21.72 ± 0.20^f^	28.79 ± 1.34^b^
	MeOH	12.11	72.50 ± 0.63^a^	43.77 ± 1.09^a^
	MeOH/water (80%)	14.26	72.26 ± 0.39^a^	23.69 ± 0.19^c^
	Aqueous	16.70	69.16 ± 0.56^b^	18.04 ± 0.25^d^
Roots	Hexane	3.81	19.58 ± 0.04^g^	2.13 ± 0.10^g^
	DCM	1.75	39.17 ± 0.58^f^	8.70 ± 0.60^e^
	MeOH	10.26	44.27 ± 0.11^e^	8.75 ± 0.48^e^
	MeOH/water (80%)	12.04	50.61 ± 0.40^c^	3.33 ± 0.06^g^
	Aqueous	11.45	45.50 ± 0.24^d^	2.52 ± 0.02^g^

a Values are reported as mean ± SD. DCM: dichloromethane; MeOH: methanol; TPC: total phenolic content; TFC: total flavonoid content; GAE: gallic acid equivalent; RE: rutin equivalent. Different letters indicate significant differences in the extracts (*p* < 0.05).

### 2.2. Profile of bioactive compounds

The total phenolic and flavonoid contents were determined using the Folin–Ciocalteu and aluminium chloride (AlCl_3_) assays, respectively.^21,22^ Results were expressed as gallic acid (mg GAEs per g extract) and rutin equivalents (mg REs per g extract) for respective assays.

Chromatographic separation was accomplished with a Dionex Ultimate 3000RS UHPLC instrument, equipped with Thermo Accucore C18 (100 mm × 2.1 mm i. d., 2.6 μm) analytical column for separation of compounds. Water (A) and methanol (B) containing 0.1% formic acid were employed as mobile phases, respectively. The total run time was 70 minutes, the elution profile and all exact analytical conditions have been published.^23^

### 2.3. Determination of antioxidant and enzyme inhibitory effects

The metal chelating, phosphomolybdenum, ferric reducing antioxidant power (FRAP), cupric reducing antioxidant capacity (CUPRAC), 2,2′-azino-bis(3-ethylbenzthiazoline-6-sulfonic acid) (ABTS), and 2,2-diphenyl-1-picrylhydrazyl (DPPH) activities of the extracts (0.5–5 mg mL^−1^) were assessed following the methods described by Grochowski *et al.*^24^ The antioxidant activities were reported as trolox equivalents, whereas ethylenediaminetetraacetic acid (EDTA) was used for metal chelating assay. The possible inhibitory effects of the extracts (0.5–5 mg mL^−1^) against cholinesterases (by Ellman's method), tyrosinase, α-amylase and α-glucosidase were evaluated using standard *in vitro* bio-assays.^24^ To provide comparison with standard antioxidants and inhibitors, IC_50_ values were also given (this is extract concentration required for scavenging 50% of radicals, ferrous ion-ferrozine and enzyme inhibitory assays; this is effective concentration at which the absorbance was 0.5 for CUPRAC, FRAP and PBD assays).

### 2.4. Antimicrobial evaluation

In this study totally twelve microorganisms (eleven bacteria and one yeast) were used to elucidate of antimicrobial potential of *S. ceratophylla* extracts. Standard microorganisms were obtained from Microbiology Research Laboratory of Vocational School of Health Services, Selcuk University. Broth micro dilution method was conducted for antimicrobial activity of extracts according to Balouiri *et al.*^25^

Briefly, 96-well plates were loaded with 100 μL Mueller Hinton Broth medium. Then 100 μL S. *ceratophylla* extracts were transferred to first well of the plate and serial dilution was done by transferring of 100 μL volume mixture *via* multichannel pipette. When the extract-medium mixture was ready then fresh microorganism inoculum prepared from 0.5 Mc Farland turbidity and final concentration 5 × 10^5^ were added to each well. Plates were sealed and incubated in an incubator at 37 °C for 18–24 hours. Gentamicin was used as positive control. After incubation period 20 μL of 2,3,5 tri phenyl tetrazolium chloride solution (0.5%) loaded to each well for detecting of minimum inhibitory concentration (MIC) of *S. ceratophylla* extracts. The MIC is the lowest concentration of antimicrobial agent that completely inhibits growth of the organism in tubes or microdilution wells as detected by the unaided eye.^26^

### 2.5. Cell culture

The human hepatocarcinoma HepG2 cells and murine bone marrow stromal S17 cells were kindly provided by the Centre for Molecular and Structural Biomedicine of Biomedical and Molecular BME, University of Algarve, Portugal), while mouse melanoma B16 4A5 cells was purchased from Sigma-Aldrich (Germany). All cell lines were cultured in Dulbecco's Modified Eagle medium (DMEM) supplemented with foetal bovine serum (10%), l-glutamine (2 mM, 1%), and penicillin (50 U mL^−1^)/streptomycin (50 μg mL^−1^) (1%), and kept under a humidified atmosphere at 37 °C and 5% CO_2_.

### 2.6. Determination of cellular viability and selectivity

Cells were plated in 96-well plates at 5 × 10^3^ cells per well (HepG2 and S17) and 2 × 10^3^ cells per well (B16 4A5). After a 24 h incubation, cells were treated with the samples at the concentration of 100 μg mL^−1^ for 72 h. Cells incubated with DMSO at 0.5% (the highest DMSO concentration used in the test wells) were used as control. The cellular viability was determined by the MTT (3-(4,5-dimethylthiazol-2-yl)-2,5-diphenyltetrazolium bromide) test, as described formerly.^27^ The percentage of viable cells was calculated relative to the control (DMSO, 0.5%). Selectivity index (SI) was calculated by the formula SI = CT/CNT, where CT and CNT stands for the cytotoxicity of the extract towards tumoral and non-tumoral cell lines, respectively.^28^

### 2.7. Data analysis

Statistical calculations were done using Xlstat 2018 and R v 3.5.1 softwares. Firstly, the one-way ANOVA with Tukey post-hoc test was performed for comparisons among samples. Pearson correlation coefficients were calculated among total bioactive compounds and biological activities. Afterwards, the biological activities dataset was analysed by supervised Partial Least Square Discriminant Analysis PLS-DA. The accuracy of model was recorded by calculating the AUC average. Finally, line plot was used following one-way ANOVA to investigate the effect of extraction solvents on the biological activities of each studied parts respectively.

## 3. Results and discussion

### 3.1. Total bioactive compounds and phytochemical composition

Plants and herbs are known to be abounded with scads of phytochemicals possessing medicinal properties such as anti-inflammatory, anticancer, and antioxidant, to name a few.^29^ The prepared aqueous, hexane, DCM, hydro-methanolic (80%) and methanolic root and aerial part extracts were evaluated for their total phenolic and flavonoid content using colorimetric methods. Results obtained are summarized in Table 1. Upon comparison between the different extracts, hexane root and aerial extracts were found to yield the least amount of phenolic and flavonoids. The same outcomes were reported in previous studies whereby hexane solvent extracted the least amount of phenolic and flavonoid content.^30–32^ The methanolic aerial extract possessed the highest phenolic (72.50 ± 0.63 mg GAE per g) and flavonoid content (43.77 ± 1.09 mg RE per g). In terms of roots, phenolic content was higher in the hydro-methanolic extract (50.61 ± 0.40 mg GAE per g) in contrast to the methanolic extract (44.27 ± 0.11 mg GAE per g). It can be said that phenolic and flavonoid compounds were better extracted in hydro-methanol and methanol solvents compared to the other extraction solvents.

**Fig. 1:**
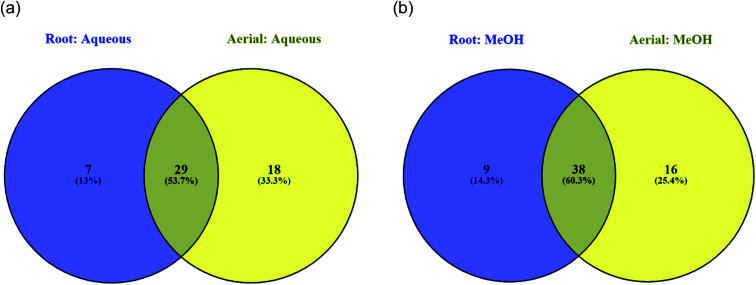
Venn diagrams displaying common compounds between different (a) aqueous (b) methanolic extracts.

**Fig. 2:**
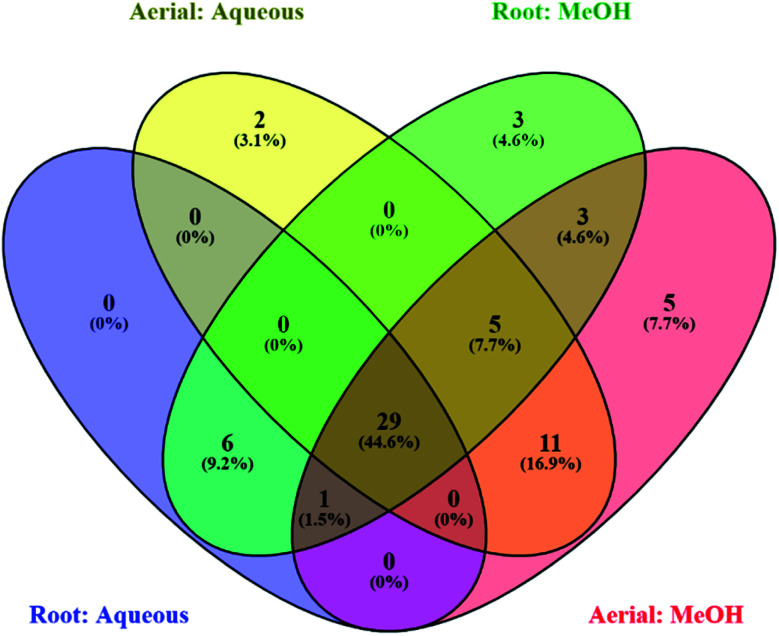
Venn diagram showing number of common compounds found in all four analysed extracts (methanolic root and aerial, aqueous root and aerial).

The LC-MS/MS analysis allowed the characterization of the chemical composition of all the studied extracts obtained from *S. ceratophylla*. In total, 54 major compounds occurring in the aerial methanolic extract were detected, 47 in methanolic root, 48 in aqueous aerial and 37 in aqueous root extracts. The detailed chromatographic results are given Tables 2–5. Twenty-nine compounds were found in common between the aqueous root and aerial extracts (Fig. 1a) while 38 were common between methanolic root and aerial extracts (Fig. 1b). Fig. 2 shows that a total of 29 phytochemicals were found in common in all four analysed extracts (methanolic root and aerial, aqueous root and aerial).

**Table 2:** Chemical composition of aerial parts-MeOH

No.	Name	Formula	Rt	[M + H]^+^	[M − H]^−^	Fragment 1	Fragment 2	Fragment 3	Fragment 4	Fragment 5
1^a^	Gallic acid (3,4,5-trihydroxybenzoic acid)	C7H6O5	2.64		169.01370	125.0230	97.0281	69.0331		
2	Dihydroxybenzoic acid	C7H6O4	5.50		153.01879	123.0437	109.0281	108.0202	81.0331	
3	Pantothenic acid	C9H17NO5	6.06	220.11850		202.1079	184.0973	174.1133	116.0346	90.0556
4	Caftaric acid (2-*O*-Caffeoyltartaric acid)	C13H12O9	8.50		311.04031	179.0340	149.0080	135.0440	87.0072	
5	Dihydroxycoumarin-*O*-hexoside	C15H16O9	12.85	331.15455		179.0342	151.0390	133.0284	123.0444	85.0291
6	Kynurenic acid	C10H7NO3	13.80	190.05042		162.0552	144.0444	116.0500	89.0392	
7	Caffeic acid	C9H8O4	15.12		179.03444	135.0439	107.0489			
8	Unidentified alkaloid	C10H11NO3	16.17	194.08172		166.0865	136.0760	108.0449	87.0447	80.0502
9	Naringenin-6,8-di-*C*-glucoside	C27H32O15	17.31		595.16630	505.1357	475.1238	415.1028	385.0929	355.0821
10	Phaselic acid (2-*O*-Caffeoylmalic acid)	C13H12O8	18.62		295.04540	179.0340	135.0439	133.0130	115.0022	71.0122
11	4-*O*-Feruloylquinic acid	C17H20O9	18.93		367.10291	193.0499	173.0444	134.0360	93.0330	
12	Loliolide	C11H16O3	19.99	197.11777		179.1070	161.0963	135.1171	133.1015	107.0860
13	Rosmarinic acid-di-*O*-hexoside	C30H36O18	22.30		683.18234	521.1315	359.0995	323.0777	197.0449	179.0340
14	Luteolin-*O*-glucuronide isomer 1	C21H18O12	22.49		461.07201	285.0407	217.0501	199.0396	151.0024	133.0280
15	Luteolin-*O*-hexoside isomer 1	C21H20O11	22.61		447.09274	327.0501	285.0407	284.0329	256.0376	151.0025
16	Luteolin-*O*-glucuronide isomer 2	C21H18O12	22.71		461.07201	285.0406	217.0500	199.0393	151.0024	133.0279
17	Luteolin-7-*O*-glucoside (cynaroside)	C21H20O11	22.86		447.09274	327.0507	285.0407	284.0330	256.0381	151.0026
18	Rosmarinic acid-*O*-hexoside	C24H26O13	23.38		521.12952	359.0730	323.0772	197.0448	179.0340	161.0232
19	Methoxy-tetrahydroxy(iso)flavone-*O*-glucuronide	C22H20O13	23.40		491.08257	315.0513	300.0277	272.0327	151.0024	113.0230
20	Apigenin-*O*-glucuronide	C21H18O11	24.36		445.07709	269.0456	225.0554	175.0237	117.0332	113.0230
21^a^	Cosmosiin (Apigenin-7-*O*-glucoside)	C21H20O10	24.44	433.11347		271.0603	153.0183	119.0501		
22	Rosmarinic acid (labiatenic acid)	C18H16O8	24.65		359.07670	197.0449	179.0340	161.0232	135.0439	133.0283
23	Methyl caffeate	C10H10O4	24.67	195.06574		163.0392	145.0287	135.0444	117.0339	89.0392
24	Chrysoeriol-7-*O*-glucuronide	C22H20O12	24.82		475.08766	299.0562	284.0329	256.0376		
25	Apigenin-*O*-hexoside	C21H20O10	24.89		431.09782	311.0562	269.0456	268.0377	151.0021	117.0336
26	Luteolin-*O*-hexoside isomer 2	C21H20O11	25.10		447.0974	285.0407	284.0330	255.0297	151.0024	133.0279
27	*N*-trans-feruloyltyramine	C18H19NO4	25.12	314.13924		194.0816	177.0548	149.0600	145.0286	121.0651
28	Abscisic acid	C15H20O4	25.75		263.12834	219.1385	204.1151	201.1281	152.0831	151.0752
29	Martynoside or isomer	C31H40O15	26.20		651.22890	475.1822	193.0500	175.0390	160.0154	134.0361
30	Pentahydroxy(iso)flavone	C15H10O7	26.26		301.03483	273.0401	257.0444	151.0023	107.0121	
31	3-*O*-Methylrosmarinic acid	C19H18O8	26.57		373.09235	197.0449	179.0340	175.0390	160.0154	135.0439
32	Dihydroactinidiolide	C11H16O2	27.07	181.12286		163.1119	145.1015	135.1171	121.1016	107.0860
33	Methoxy-trihydroxy(iso)flavone isomer 1	C16H12O6	28.06		299.05556	284.0328	283.0252	256.0378	228.0422	227,0345
34^a^	Luteolin (3′,4′,5,7-Tetrahydroxyflavone)	C15H10O6	28.37		285.03991	217.0495	199.0393	175.0387	151.0024	133.0282
35	*N*1,*N*5,*N*10-Tricoumaroylspermidine	C34H37N3O6	29.46		582.26042	462.2038	436.2245	342.1458	145.0283	119.0488
36	Apigenin (4′,5,7-Trihydroxyflavone)	C15H10O5	30.22		269.04500	225.0547	201.0557	151.0024	149.0232	117.0330
37	Chrysoeriol (3′-methoxy-4′,5,7-trihydroxyflavone)	C16H12O6	30.44		299.05556	284.0329	283.0251	256.0376	227.0344	151.0018
38	Dihydrololiolide	C11H18O3	30.50	199.13342		181.1226	163.1119	135.1172	111.0445	107.0860
39	Methoxy-tetrahydroxy(iso)flavone	C16H12O7	30.54		315.05048	300.0277	272.0326	227.0335	151.0026	149.0233
40	Undecanedioic acid	C11H20O4	31.32		215.12834	197.1176	153.1272	125.0959	57.0332	
41	Dihydroxy-trimethoxy(iso)flavone	C18H16O7	31.83	345.09743		330.0735	329.0663	315.0495	312.0631	284.0682
42	Dihydroxy-dimethoxy(iso)flavone	C17H14O6	32.42	315.08686		300.0632	272.0678	257.0447	229.0487	
43	Methoxy-trihydroxy(iso)flavone isomer 2	C16H12O6	33.02		299.05556	284.0328	283.0237	256.0375	227.0346	151.0030
44	Hydroxy-tetramethoxy(iso)flavone	C19H18O7	33.31	359.11308		344.0891	343.0810	326.0790	315.0862	298.0838
45	Dodecanedioic acid	C12H22O4	33.75		229.14399	211.1334	185.1539	167.1431		
46^a^	Genkwanin (4′,5-dihydroxy-7-methoxyflavone)	C16H12O5	35.05	285.07630		270.0525	242.0574	213.0543	167.0341	119.0493
47	Hydroxy-trimethoxy(iso)flavone	C18H16O6	35.34	329.10252		314.0788	313.0701	299.0547	296.0683	268.0731
48	Apigenin-4′,7-dimethyl ether (4′,7-dimethoxy-5-hydroxyflavone)	C17H14O5	38.71	299.09195		284.0682	256.0731	167.0338	133.0649	
49	Stearidonic acid	C18H28O2	40.13		275.20111	231.2107	177.1633	59.0124		
50	Hydroxyoctadecatrienoic acid	C18H30O3	40.21		293.21167	275.2020	235.1700	231.2117	171.1018	121.1008
51	Unidentified terpene 1	C20H30O2	41.92	303.23241		285.2215	267.2123	257.2264	247.1695	201.1644
52	Unidentified terpene 2	C30H48O4	43.42	473.36309		455.3521	437.3416	419.3310	401.3207	359.2582
53	Unidentified terpene 3	C30H48O4	43.59	473.36309		455.3523	437.3418	419.3314	401.3216	359.2582
54	Unidentified terpene 4	C30H48O4	44.26	473.36309		455.3520	437.3418	419.3313	401.3202	109.1016

a Confirmed by standard.

**Table 3:** Chemical composition of aerial parts-aqueous

No.	Name	Formula	Rt	[M + H]^+^	[M − H]^−^	Fragment 1	Fragment 2	Fragment 3	Fragment 4	Fragment 5
1	Dihydroxybenzoic acid	C7H6O4	5.47		153.01879	123.0439	109.0281	108.0203	81.0331	
2	Pantothenic acid	C9H17NO5	6.03	220.11850		202.1088	184.0973	174.1128	116.0347	90.0555
3	Caftaric acid (2-*O*-Caffeoyltartaric acid)	C13H12O9	8.48		311.04031	179.0340	149.0079	135.0439	87.0072	
4	Kynurenic acid	C10H7NO3	13.77	190.05042		162.0552	144.0448	116.0497	89.0394	
5	Caffeic acid	C9H8O4	15.10		179.03444	135.0439	107.0489			
6	Unidentified alkaloid	C10H11NO3	16.15	194.08172		166.0865	136.0760	108.0449	87.0447	80.0502
7	Naringenin-6,8-di-*C*-glucoside	C27H32O15	17.28		595.16630	505.1334	475.1242	415.1036	385.0932	355.0826
8	Phaselic acid (2-*O*-Caffeoylmalic acid)	C13H12O8	18.60		295.04540	179.0340	135.0440	133.0130	115.0022	71.0122
9	Loliolide	C11H16O3	19.97	197.11777		179.1070	161.0963	135.1172	133.1016	107.0861
10	Rosmarinic acid-di-*O*-hexoside	C30H36O18	22.28		683.18234	521.1299	359.0994	323.0775	197.0449	179.0340
11	Rosmarinic acid-*O*-hexoside isomer 1	C24H26O13	22.37		521.12952	359.0753	323.0766	197.0449	179.0340	161.0232
12	Luteolin-*O*-glucuronide isomer 2	C21H18O12	22.65		461.07201	285.0407	217.0501	199.0389	151.0024	133.0280
13	Luteolin-7-*O*-glucoside (cynaroside)	C21H20O11	22.84		447.09274	327.0524	285.0407	284.0329	256.0371	151.0023
14	Rosmarinic acid-*O*-hexoside isomer 2	C24H26O13	23.36		521.12952	359.0772	323.0775	197.0448	179.0340	161.0232
15	Methoxy-tetrahydroxy(iso)flavone-*O*-glucuronide	C22H20O13	23.39		491.08257	315.0514	300.0278	272.0326	151.0024	113.0230
16^a^	Cosmosiin (apigenin-7-*O*-glucoside)	C21H20O10	24.45	433.11347		271.0604	153.0186	119.0491		
17	Apigenin-*O*-glucuronide	C21H18O11	24.49		445.07709	269.0457	225.0549	175.0235	117.0332	113.0230
18	Methyl caffeate	C10H10O4	24.63	195.06574		163.0392	145.0287	135.0444	117.0339	89.0392
19	Rosmarinic acid (labiatenic acid)	C18H16O8	24.66		359.07670	197.0449	179.0340	161.0232	135.0439	133.0282
20	Chrysoeriol-7-*O*-glucuronide	C22H20O12	24.82		475.08766	299.0562	284.0328	256.0385		
21	*N-trans*-Feruloyltyramine	C18H19NO4	25.12	314.13924		194.0816	177.0548	149.0600	145.0286	121.0651
22	Luteolin-*O*-hexoside isomer 2	C21H20O11	25.13		447.09274	285.0407	284.0328	255.0298	151.0025	133.0280
23	Abscisic acid	C15H20O4	25.77		263.12834	219.1385	204.1150	201.1279	152.0830	151.0752
24	Martynoside or isomer	C31H40O15	26.22		651.22890	475.1835	193.0499	175.0390	160.0154	134.0362
25	Pentahydroxy(iso)flavone	C15H10O7	26.28		301.03483	273.0401	257.0452	151.0025	107.0126	
26	3-*O*-Methylrosmarinic acid	C19H18O8	26.57		373.09235	197.0449	179.0340	175.0389	160.0153	135.0439
27	Dihydroactinidiolide	C11H16O2	27.08	181.12286		163.1120	145.1014	135.1172	121.1016	107.0860
28	Martynoside or isomer	C31H40O15	27.56		651.22890	475.1806	193.0501	175.0389	160.0152	134.0358
29	Methoxy-trihydroxy(iso)flavone isomer 1	C16H12O6	28.09		299.05556	284.0329	283.0256	256.0375	228.0427	227.0342
30^a^	Luteolin (3′,4′,5,7-Tetrahydroxyflavone)	C15H10O6	28.38		285.03991	217.0494	199.0392	175.0392	151.0024	133.0282
31	*N*1,*N*5,*N*10-Tricoumaroylspermidine	C34H37N3O6	29.48		582.26042	462.2035	436.2205	342.1466	145.0282	119.0488
32	Apigenin (4′,5,7-Trihydroxyflavone)	C15H10O5	30.24		269.04500	225.0550	201.0555	151.0024	149.0233	117.0331
33	Chrysoeriol (3′-methoxy-4′,5,7-trihydroxyflavone)	C16H12O6	30.44		299.05556	284.0329	283.0245	256.0378	227.0351	151.0027
34	Dihydrololiolide	C11H18O3	30.49	199.13342		181.1226	163.1119	135.1171	111.0445	107.0861
35	Undecanedioic acid	C11H20O4	31.32		215.12834	197.1177	153.1273	125.0961	57.0333	
36	Dihydroxy-trimethoxy(iso)flavone	C18H16O7	31.82	345.09743		330.0737	329.0654	315.0501	312.0631	284.0682
37	Dihydroxy-dimethoxy(iso)flavone	C17H14O6	32.41	315.08686		300.0631	272.0682	257.0448	229.0487	
38	Methoxy-trihydroxy(iso)flavone isomer 2	C16H12O6	33.03		299.05556	284.0329	283.0239	256.0371	227.0346	151.0031
39	Hydroxy-tetramethoxy(iso)flavone	C19H18O7	33.31	359.11308		344.0887	343.0818	326.0790	315.0881	298.0839
40	Dodecanedioic acid	C12H22O4	33.76		229.14399	211.1334	185.1530	167.1430		
41^a^	Genkwanin (4′,5-dihydroxy-7-methoxyflavone)	C16H12O5	35.04	285.07630		270.0526	242.0577	213.0543	167.0342	119.0494
42	Hydroxy-trimethoxy(iso)flavone	C18H16O6	35.33	329.10252		314.0786	313.0719	299.0546	296.0682	268.0732
43	Apigenin-4′,7-dimethyl ether (4′,7-dimethoxy-5-hydroxyflavone)	C17H14O5	38.71	299.09195		284.0683	256.0732	167.0344	133.0654	
44	Stearidonic acid	C18H28O2	40.15		275.20111	231.2120	177.1633	59.0126		
45	Hydroxyoctadecatrienoic acid	C18H30O3	40.22		293.21167	275.2019	235.1700	231.2110	171.1016	121.1008
46	Unidentified terpene 1	C20H30O2	41.94	303.23241		285.2216	267.2104	257.2267	247.1689	201.1644
47	Unidentified terpene 2	C30H48O4	43.42	473.36309		455.3527	437.3422	419.3322	401.3216	359.2585
48	Unidentified terpene 4	C30H48O4	44.30	473.36309		455.3526	437.3422	419.3319	401.3214	109.1017

a Confirmed by standard.

**Table 4:** Chemical composition of root-MeOH

No.	Name	Formula	Rt	[M + H]^+^	[M − H]^−^	Fragment 1	Fragment 2	Fragment 3	Fragment 4	Fragment 5
1^a^	Gallic acid (3,4,5-trihydroxybenzoic acid)	C7H6O5	2.69		169.01370	125.0230	97.0279	69.0331		
2	Dihydroxybenzoic acid	C7H6O4	5.55		153.01879	123.0438	109.0281	108.0203	81.0331	
3	Pantothenic acid	C9H17NO5	6.17	220.11850		202.1077	184.0973	174.1124	116.0346	90.0555
4	Caftaric acid (2-*O*-Caffeoyltartaric acid)	C13H12O9	8.56		311.04031	179.0341	149.0080	135.0439	87.0070	
5	Salicylic acid-2-*O*-glucoside	C13H16O8	13.50		299.07670	137.0232	113.0230	93.0330	85.0280	71.0123
6	Kynurenic acid	C10H7NO3	13.82	190.05042		162.0552	144.0447	116.0498	89.0392	
7	Caffeoylhexose	C15H18O9	14.88		341.08726	179.0340	135.0440	107.0486	89.0229	71.0124
8	Caffeic acid	C9H8O4	15.13		179.03444	135.0439	107.0489			
9	Phaselic acid (2-*O*-Caffeoylmalic acid)	C13H12O8	18.61		295.04540	179.0341	135.0440	133.0130	115.0022	71.0122
10	4-*O*-Feruloylquinic acid	C17H20O9	18.92		367.10291	193.0498	173.0445	134.0361	93.0330	
11	Loliolide	C11H16O3	19.98	197.11777		179.1070	161.0963	135.1172	133.1015	107.0861
12	Rosmarinic acid-di-*O*-hexoside	C30H36O18	22.28		683.18234	521.1306	359.1000	323.0775	197.0449	179.0341
13	Luteolin-*O*-glucuronide isomer 2	C21H18O12	22.74		461.07201	285.0407	217.0495	199.0393	151.0025	133.0281
14	Luteolin-7-*O*-glucoside (cynaroside)	C21H20O11	22.83		447.09274	327.0510	285.0408	284.0330	256.0377	151.0023
15	Rosmarinic acid-*O*-hexoside	C24H26O13	23.38		521.12952	359.0770	323.0774	197.0450	179.0341	161.0233
16^a^	Cosmosiin (apigenin-7-*O*-glucoside)	C21H20O10	24.46	433.11347		271.0603	153.0184	119.0495		
17	Apigenin-*O*-glucuronide	C21H18O11	24.49		445.07709	269.0457	225.0544	175.0235	117.0330	113.0230
18	Rosmarinic acid (labiatenic acid)	C18H16O8	24.64		359.07670	197.0450	179.0341	161.0233	135.0440	133.0283
19	Methyl caffeate	C10H10O4	24.65	195.06574		163.0392	145.0287	135.0444	117.0339	89.0391
20	Luteolin-*O*-hexoside isomer 2	C21H20O11	25.10		447.09274	285.0408	284.0336	255.0304	151.0025	133.0283
21	*N-trans*-Feruloyltyramine	C18H19NO4	25.12	314.13924		194.0820	177.0549	149.0602	145.0287	121.0652
22	Martynoside or isomer	C31H40O15	26.21		651.22890	475.1812	193.0500	175.0390	160.0154	134.0361
23	3-*O*-Methylrosmarinic acid	C19H18O8	26.57		373.09235	197.0449	179.0340	175.0390	160.0154	135.0439
24	Dihydroactinidiolide	C11H16O2	27.08	181.12286		163.1120	145.1016	135.1172	121.1016	107.0861
25	Methoxy-trihydroxy(iso)flavone isomer 1	C16H12O6	28.07		299.05556	284.0330	283.0243	256.0378	228.0424	227.0351
26^a^	Luteolin (3′,4′,5,7-Tetrahydroxyflavone)	C15H10O6	28.36		285.03991	217.0499	199.0395	175.0390	151.0025	133.0282
27	Apigenin (4′,5,7-Trihydroxyflavone)	C15H10O5	30.22		269.04500	225.0553	201.0557	151.0026	149.0233	117.0331
28	Chrysoeriol (3′-methoxy-4′,5,7-trihydroxyflavone)	C16H12O6	30.43		299.05556	284.0329	283.0255	256.0377	227.0352	151.0023
29	Undecanedioic acid	C11H20O4	31.30		215.12834	197.1178	153.1273	125.0959	57.0332	
30	Dihydroxy-trimethoxy(iso)flavone	C18H16O7	31.81	345.09743		330.0736	329.0659	315.0503	312.0631	284.0682
31	Dihydroxy-dimethoxy(iso)flavone	C17H14O6	32.42	315.08686		300.0633	272.0681	257.0439	229.0487	
32	Methoxy-trihydroxy(iso)flavone isomer 2	C16H12O6	32.99		299.05556	284.0329	283.0252	256.0375	227.0344	151.0029
33	Dodecanedioic acid	C12H22O4	33.75		229.14399	211.1334	185.1556	167.1431		
34^a^	Genkwanin (4′,5-dihydroxy-7-methoxyflavone)	C16H12O5	35.04	285.07630		270.0526	242.0576	213.0552	167.0342	119.0495
35	Hydroxy-trimethoxy(iso)flavone	C18H16O6	35.32	329.10252		314.0788	313.0718	299.0540	296.0683	268.0732
36	Unidentified terpene 5	C20H30O3	36.08	319.22732		301.2169	291.2325	289.2166	277.1802	165.0914
37	Unidentified terpene 6	C20H26O4	38.49	331.19094		313.1800	295.1698	267.1746	229.1226	211.1121
38	Apigenin-4′,7-dimethyl ether (4′,7-dimethoxy-5-hydroxyflavone)	C17H14O5	38.69	299.09195		284.0682	256.0732	167.0340	133.0650	
39	Unidentified terpene 7	C21H28O4	39.90	345.20658		327.1961	313.1799	295.1696	267.1746	229.1226
40	Unidentified terpene 8	C20H26O4	40.00	331.19094		313.1802	295.1700	267.1744	229.1226	211.1121
41	Hydroxyoctadecatrienoic acid	C18H30O3	40.21		293.21167	275.2020	235.1692	231.2117	171.1012	121.1012
42	Unidentified terpene 9	C21H28O4	41.96	345.20658		327.1966	313.1802	295.1700	267.1746	229.1226
43	Viridoquinone	C20H24O2	42.23	297.18546		279.1748	269.1896	239.1433	237.1277	197.0966
44	Unidentified terpene 2	C30H48O4	43.39	473.36309		455.3525	437.3420	419.3312	401.3196	359.2586
45	Unidentified terpene 3	C30H48O4	43.56	473.36309		455.3528	437.3425	419.3318	401.3213	359.2586
46	Unidentified terpene 4	C30H48O4	44.25	473.36309		455.3527	437.3424	419.3318	401.3228	109.1017
47	Unidentified terpene 10	C30H50O2	46.23	443.38891		425.3799	407.3697	217.1951	203.1799	191.1799

a Confirmed by standard.

**Table 5:** Chemical composition of roots-aqueous

No.	Name	Formula	Rt	[M + H]^+^	[M − H]^−^	Fragment 1	Fragment 2	Fragment 3	Fragment 4	Fragment 5
1	Dihydroxybenzoic acid	C7H6O4	5.51		153.01879	123.0438	109.0280	108.0203	81.0332	
2	Pantothenic acid	C9H17NO5	6.15	220.11850		202.1077	184.0973	174.1128	116.0347	90.0556
3	Caftaric acid (2-*O*-Caffeoyltartaric acid)	C13H12O9	8.53		311.04031	179.0340	149.0079	135.0439	87.0071	
4	Salicylic acid-2-*O*-glucoside	C13H16O8	13.49		299.07670	137.0232	113.0230	93.0330	85.0279	71.0123
5	Kynurenic acid	C10H7NO3	13.80	190.05042		162.0552	144.0446	116.0499	89.0393	
6	Caffeoylhexose	C15H18O9	14.88		341.08726	179.0340	135.0439	107.0486	89.0228	71.0123
7	Caffeic acid	C9H8O4	15.13		179.03444	135.0439	107.0489			
8	Phaselic acid (2-*O*-Caffeoylmalic acid)	C13H12O8	18.61		295.04540	179.0340	135.0439	133.0130	115.0022	71.0122
9	Loliolide	C11H16O3	19.99	197.11777		179.1070	161.0963	135.1172	133.1016	107.0861
10	Rosmarinic acid-di-*O*-hexoside	C30H36O18	22.30		683.18234	521.1307	359.1003	323.0774	197.0449	179.0340
11	Luteolin-*O*-glucuronide isomer 2	C21H18O12	22.73		461.07201	285.0406	217.0501	199.0387	151.0025	133.0280
12	Luteolin-7-*O*-glucoside (cynaroside)	C21H20O11	22.82		447.09274	327.0513	285.0407	284.0329	256.0371	151.0023
13	Rosmarinic acid-*O*-hexoside	C24H26O13	23.38		521.12952	359.0762	323.0773	197.0449	179.0340	161.0232
14^a^	Cosmosiin (Apigenin-7-*O*-glucoside)	C21H20O10	24.45	433.11347		271.0603	153.0183	119.0496		
15	Apigenin-*O*-glucuronide	C21H18O11	24.48		445.07709	269.0457	225.0553	175.0238	117.0327	113.0230
16	Rosmarinic acid (labiatenic acid)	C18H16O8	24.67		359.07670	197.0449	179.0340	161.0232	135.0439	133.0283
17	Methyl caffeate	C10H10O4	24.68	195.06574		163.0392	145.0287	135.0444	117.0339	89.0391
18	*N-trans*-Feruloyltyramine	C18H19NO4	25.11	314.13924		194.0822	177.0547	149.0598	145.0286	121.0653
19	Martynoside or isomer	C31H40O15	26.21		651.22890	475.1839	193.0501	175.0390	160.0154	134.0361
20	3-*O*-Methylrosmarinic acid	C19H18O8	26.57		373.09235	197.0449	179.0340	175.0390	160.0154	135.0439
21	Dihydroactinidiolide	C11H16O2	27.07	181.12286		163.1119	145.1014	135.1172	121.1015	107.0860
22	Martynoside or isomer	C31H40O15	27.56		651.22890	475.1825	193.0500	175.0390	160.0154	134.0361
23^a^	Luteolin (3′,4′,5,7-Tetrahydroxyflavone)	C15H10O6	28.38		285.03991	217.0509	199.0388	175.0390	151.0023	133.0282
24	Apigenin (4′,5,7-Trihydroxyflavone)	C15H10O5	30.23		269.04500	225.0549	201.0553	151.0024	149.0229	117.0332
25	Undecanedioic acid	C11H20O4	31.31		215.12834	197.1177	153.1273	125.0959	57.0332	
26	Dihydroxy-dimethoxy(iso)flavone	C17H14O6	32.43	315.08686		300.0630	272.0682	257.0434	229.0487	
27	Dodecanedioic acid	C12H22O4	33.76		229.14399	211.1334	185.1533	167.1430		
28^a^	Genkwanin (4′,5-dihydroxy-7-methoxyflavone)	C16H12O5	35.05	285.07630		270.0528	242.0575	213.0552	167.0342	119.0497
29	Hydroxy-trimethoxy(iso)flavone	C18H16O6	35.34	329.10252		314.0787	313.0709	299.0543	296.0683	268.0732
30	Unidentified terpene 5	C20H30O3	36.07	319.22732		301.2164	291.2324	289.2161	277.1803	165.0913
31	Unidentified terpene 6	C20H26O4	38.51	331.19094		313.1800	295.1696	267.1747	229.1226	211.1121
32	Apigenin-4′,7-dimethyl ether (4′,7-dimethoxy-5-hydroxyflavone)	C17H14O5	38.72	299.09195		284.0682	256.0732	167.0346	133.0650	
33	Unidentified terpene 8	C20H26O4	40.00	331.19094		313.1799	295.1694	267.1748	229.1227	211.1121
34	Hydroxyoctadecatrienoic acid	C18H30O3	40.22		293.21167	275.2019	235.1702	231.2116	171.1014	121.1009
35	Viridoquinone	C20H24O2	42.24	297.18546		279.1748	269.1897	239.1433	237.1276	197.0965
36	Unidentified terpene 2	C30H48O4	43.42	473.36309		455.3525	437.3422	419.3309	401.3203	359.2583
37	Unidentified terpene 4	C30H48O4	44.28	473.36309		455.3522	437.3423	419.3311	401.3228	109.1017

a Confirmed by standard.

### 3.2. Antioxidant activities

Six methods namely DPPH, ABTS, FRAP, CUPRAC, phosphomolybdenum and metal chelating were used to assess the antioxidant activities of the prepared extracts. Table 6 details the data gathered in this work. The remarkably high antioxidant activity was found to be distributed among the hydro-methanolic, methanolic and aqueous extracts while the hexane extracts exhibited the lowest antioxidant activity with all methods irrespective of the plant part used. For instance, the hydro-methanolic aerial extract showed the maximum DPPH radical scavenging activity (193.40 ± 0.27 mg TE per g) and the highest reducing potential towards copper(ii) (377.93 ± 2.38 mg TE per g). Among the different root extracts analysed, the hydro-methanolic sample revealed to be the most potent ABTS radical scavenger (116.50 ± 1.65 mg TE per g) and displayed the highest reducing potential with both CUPRAC (250.03 ± 2.65 mg TE per g) and FRAP (142.00 ± 0.14 mg TE per g) assays. Similar findings were recorded in previous work showing that hydro-alcoholic extracts possessed substantially higher antioxidant activity compared to other extracts derived from low polarity solvents.^33,34^ In a study conducted by Orhan *et al.*,^15^ the methanolic extract displayed a high percentage inhibition of 84.8 ± 1.11 against DPPH radicals, corroborating our results. The total antioxidant capacity of the aerial and root extracts ranged from 1.81–2.48 and 0.97–2.41 mmol TE per g, respectively. The metal chelating ability was higher with aqueous aerial (28.25 ± 0.34 mg EDTAE per g) followed by root (27.83 ± 0.49 mg EDTAE per g) extracts. Mounting evidence showed that natural products play a vital role in hindering β-amyloid fibril aggregation due to their ability to bind metal ions with high affinities.^35^

**Table 6:** Antioxidant properties of *Salvia ceratophylla* extracts^a^

Parts	Solvents	DPPH		ABTS		CUPRAC		FRAP		MCA		PBD	
		(mg TE per g)	IC_50_ (mg mL^−1^)	(mg TE per g)	IC_50_ (mg mL^−1^)	(mg TE per g)	IC_50_ (mg mL^−1^)	(mg TE per g)	IC_50_ (mg mL^−1^)	(mg TE per g)	IC_50_ (mg mL^−1^)	(mmol TE per g)	IC_50_ (mg mL^−1^)
Aerial parts	Hexane	6.47 ± 0.80^h^	>5	3.88 ± 0.34^i^	>5	48.30 ± 0.57^i^	2.68 ± 0.03^k^	23.91 ± 1.41^h^	1.97 ± 0.12^i^	na	na	1.81 ± 0.08^bc^	1.44 ± 0.06^cd^
	DCM	11.83 ± 1.37^g^	4.66 ± 0.54^h^	10.40 ± 1.38^h^	>5	79.73 ± 1.13^h^	1.62 ± 0.02^i^	31.27 ± 0.51^h^	1.50 ± 0.02^i^	na	na	2.20 ± 0.19^ab^	1.19 ± 0.11^bc^
	MeOH	188.81 ± 0.68^b^	0.29 ± 0.01^c^	125.36 ± 0.43^c^	0.60 ± 0.01^d^	324.13 ± 11.42^c^	0.40 ± 0.01^d^	172.49 ± 6.32^b^	0.27 ± 0.01^c^	17.89 ± 0.59^e^	1.15 ± 0.04^f^	2.48 ± 0.22^a^	1.06 ± 0.10^b^
	MeOH/water (80%)	193.40 ± 0.27^a^	0.28 ± 0.01^b^	155.43 ± 1.38^b^	0.48 ± 0.01^c^	377.93 ± 2.38^a^	0.34 ± 0.01^b^	217.46 ± 3.46^a^	0.22 ± 0.01^b^	19.38 ± 0.29^c^	1.06 ± 0.02^d^	2.40 ± 0.21^a^	1.09 ± 0.10^b^
	Aqueous	187.33 ± 0.86^b^	0.29 ± 0.01^c^	191.93 ± 2.42^a^	0.39 ± 0.01^b^	342.83 ± 2.43^b^	0.38 ± 0.01^c^	219.20 ± 1.72^a^	0.21 ± 0.01^b^	28.25 ± 0.34^a^	0.73 ± 0.02^b^	1.93 ± 0.09^bc^	1.36 ± 0.06^cd^
Roots	Hexane	46.13 ± 0.73^f^	1.18 ± 0.02^g^	37.03 ± 0.51^g^	2.03 ± 0.03^h^	84.29 ± 2.93^h^	1.54 ± 0.05^i^	47.72 ± 0.10^g^	0.99 ± 0.01^h^	1.63 ± 0.07^f^	>5	0.97 ± 0.05^d^	2.68 ± 0.15^e^
	DCM	80.61 ± 0.46^e^	0.68 ± 0.01^f^	92.76 ± 1.00^f^	0.81 ± 0.01^g^	183.12 ± 0.85^g^	0.71 ± 0.01^h^	98.33 ± 2.67^f^	0.48 ± 0.01^g^	23.43 ± 0.31^b^	0.87 ± 0.01^c^	2.41 ± 0.08^a^	1.08 ± 0.04^b^
	MeOH	97.60 ± 0.32^c^	0.56 ± 0.01^d^	105.25 ± 1.97^e^	0.71 ± 0.01^f^	229.95 ± 0.63^e^	0.56 ± 0.01^f^	128.91 ± 0.83^d^	0.36 ± 0.01^e^	17.91 ± 0.24^d^	1.14 ± 0.02^e^	1.81 ± 0.12^bcd^	1.45 ± 0.10^cde^
	MeOH/water (80%)	96.95 ± 0.04^c^	0.56 ± 0.01^d^	116.50 ± 1.65^d^	0.65 ± 0.01^e^	250.03 ± 2.65^d^	0.52 ± 0.01^e^	142.00 ± 0.14^c^	0.33 ± 0.01^d^	18.96 ± 0.31^c^	1.08 ± 0.02^d^	1.73 ± 0.05^cd^	1.51 ± 0.05^de^
	Aqueous	89.70 ± 1.51^d^	0.61 ± 0.01^e^	105.46 ± 0.64^e^	0.71 ± 0.01	200.52 ± 1.28^f^	0.64 ± 0.01	115.01 ± 1.97^e^	0.41 ± 0.01^f^	27.83 ± 0.49^a^	0.74 ± 0.01^b^	1.51 ± 0.14^d^	1.73 ± 0.15^e^
Standards	Trolox	—	0.05 ± 0.01^a^		0.07 ± 0.01^a^	—	0.13 ± 0.01^a^	—	0.05 ± 0.01^a^	—	nt	—	0.65 ± 0.01^a^
	EDTA	—	nt		nt	—	nt	—	nt	—	0.02 ± 0.01^a^	—	nt

a Values are reported as mean ± SD. DCM: dichloromethane; MeOH: methanol; TE: trolox equivalent; EDTAE: EDTA equivalent; MCA: metal chelating ability; PBD: phosphomolybdenum.; nt: no tested. Different letters indicate significant differences in the extracts (*p* < 0.05, the letter “a” indicates strong ability). IC_50_ (mg mL^−1^), effective concentration at which the absorbance was 0.5 for CUPRAC, FRAP and PBD assays and at which 50% of the DPPH and ABTS radicals were scavenged and the ferrous ion-ferrozine complex were inhibited.

### 3.3. Enzyme inhibitory effects

In this research work, the extracts of *S. ceratophylla* were screened for possible enzyme inhibitory effects against several non-communicable diseases including diabetes mellitus type II (α-amylase and α-glucosidase), Alzheimer's disease (AChE and BChE) and skin hyperpigmentation (tyrosinase). These aforementioned diseases were targeted since no cure has been found yet to combat such pathological disorders and the statistics presented by the World Health Organisation (WHO) is alarming. For instance, more than 420 million people have been diagnosed with diabetes^2^ and about 50 million people have dementia.^2^ Hence, searching for treatment and novel drugs should be an ongoing process. The WHO has approved drugs derived from plants to combat diabetes for various reasons, such as: (i) non-toxicity, (ii) negligible adverse effects compared to synthetic antidiabetic drugs, (iii) economically viable and, (iv) their safety has been confirmed through traditional medicine.^36^

Results obtained from the enzyme inhibitory effects of *S. ceratophylla* are shown in Table 7. All samples exhibited inhibitory activities against tyrosinase, amylase and glucosidase. Both aqueous root and aerial extracts were ineffective against cholinesterase enzymes. The petroleum ether and ethyl acetate extracts were also found inactive against BChE according to the study of Orhan *et al.*^15^ The DCM root and aerial extracts showed the highest tyrosinase (125.45 ± 1.41 and 124.68 ± 4.47 mg KAE per g, respectively) and amylase (0.76 ± 0.02 and 0.84 ± 0.02 mmol ACAE per g, respectively) activities. To the best of our knowledge, it is the first time *S. ceratophylla* was screened for tyrosinase, amylase and glucosidase activities. Therefore, comparison of our data with other work was not possible.

**Table 7:** Enzyme inhibitory properties of *Salvia ceratophylla* extracts^a^

Parts	Solvents	AChE inhibition		BChE inhibition		Tyrosinase inhibition		Amylase inhibition		Glucosidase inhibition	
		(mg GALAE per g)	IC_50_ (mg mL^−1^)	(mg GALAE per g)	IC_50_ (mg mL^−1^)	(mg KAE per g)	IC_50_ (mg mL^−1^)	(mmol ACAE per g)	IC_50_ (mg mL^−1^)	(mmol ACAE per g)	IC_50_ (mg mL^−1^)
Aerial parts	Hexane	3.78 ± 0.36^c^	0.71 ± 0.07^d^	5.65 ± 0.45^a^	1.06 ± 0.09^b^	96.32 ± 4.09^cd^	0.90 ± 0.04^de^	0.75 ± 0.05^b^	1.78 ± 0.12^c^	2.13 ± 0.01^cd^	0.55 ± 0.01^de^
	DCM	3.22 ± 0.04^d^	0.84 ± 0.01^e^	6.55 ± 1.33^a^	0.94 ± 0.19^b^	124.68 ± 4.47^a^	0.69 ± 0.02^b^	0.84 ± 0.02^a^	1.59 ± 0.03^b^	2.17 ± 0.01^c^	0.54 ± 0.01^d^
	MeOH	4.37 ± 0.27^ab^	0.62 ± 0.04^bc^	2.81 ± 0.36^b^	2.14 ± 0.25^c^	107.99 ± 8.04^bc^	0.80 ± 0.06^cd^	0.72 ± 0.03^bc^	1.85 ± 0.07^cd^	2.16 ± 0.02^c^	0.55 ± 0.01^d^
	MeOH/water (80%)	2.58 ± 0.03^e^	1.04 ± 0.01^f^	na	na	111.50 ± 4.42^abc^	0.78 ± 0.03^bcd^	0.73 ± 0.01^bc^	1.83 ± 0.03^cd^	0.24 ± 0.01^g^	4.99 ± 0.30^h^
	Aqueous	na	na	na	na	82.68 ± 8.12^de^	1.05 ± 0.11^ef^	0.14 ± 0.01^d^	>5	0.05 ± 0.01^h^	>5
Roots	Hexane	3.93 ± 0.15^bc^	0.68 ± 0.03^cd^	6.99 ± 0.42^a^	0.85 ± 0.05^b^	112.10 ± 1.73^ab^	0.77 ± 0.01^bc^	0.68 ± 0.01^c^	1.95 ± 0.01^d^	2.21 ± 0.01^bc^	0.53 ± 0.01^cd^
	DCM	4.62 ± 0.13^a^	0.58 ± 0.02^b^	na	na	125.45 ± 1.41^a^	0.69 ± 0.01^b^	0.76 ± 0.02^b^	1.76 ± 0.04^c^	2.07 ± 0.01^c^	0.57 ± 0.01^d^
	MeOH	4.17 ± 0.03^abc^	0.64 ± 0.01^bcd^	6.19 ± 0.29^a^	0.96 ± 0.04^b^	116.23 ± 7.23^ab^	0.74 ± 0.05^bc^	0.70 ± 0.01^bc^	1.90 ± 0.03^cd^	2.31 ± 0.01^a^	0.51 ± 0.01^b^
	MeOH/water (80%)	2.73 ± 0.22^de^	0.99 ± 0.08^ef^	3.67 ± 0.25^b^	1.63 ± 0.11^c^	106.56 ± 4.50^bc^	0.81 ± 0.04^cd^	0.75 ± 0.01^b^	1.77 ± 0.02^c^	1.02 ± 0.07^d^	1.16 ± 0.08^e^
	Aqueous	na	na	na	na	73.36 ± 1.85^e^	1.18 ± 0.03^f^	0.13 ± 0.01^d^	>5	0.79 ± 0.03^e^	1.49 ± 0.07^f^
Standards	Galantamine	—	0.0027 ± 0.001^a^	—	0.006 ± 0.001^a^	—	nt	—	nt	—	nt
	Kojic acid	—	nt	—	nt	—	0.09 ± 0.01^a^	—	nt	—	nt
	Acarbose	—	nt	—	nt	—	nt	—	0.86 ± 0.01^a^	—	0.76 ± 0.01^a^

a Values are reported as mean ± SD. DCM: dichloromethane; MeOH: methanol; GALAE: galantamine equivalent; KAE: kojic acid equivalent; ACAE: acarbose equivalent; na: not active.; nt: not tested. Different letters indicate significant differences in the extracts (*p* < 0.05, the letter “a” indicates strong ability). IC_50_ (mg mL^−1^), inhibition concentration at which 50% of the enzyme activities were inhibited.

### 3.4. Antimicrobial evaluation

The broth microdilution assay results were given in Table 8. According to the results obtained from test, hexane extracts of aerial parts of *S. ceratophylla* revealed MIC values ranging between 3.12–0.019 mg mL^−1^ doses. It was seen that *Sarcina lutea* was the most sensitive bacteria against to aerial hexane extract with a dose of 0.097 mg mL^−1^ MIC and followed by the *Bacillus cereus* with 0.19 MIC value. For *Citrobacter* MIC was found as 1.56 mg mL^−1^. Aerial part hexane extract had antifungal capacity at dose of 3.12 mg mL^−1^ against *Candida albicans*. While *Pseudomonas aeruginosa* was resistant to aerial part hexane extract it affected from root extracts at a concentration of 1.56 mg mL^−1^. Same as *Pseudomonas*, root extract was more effective than aerial part extract against *Staphylococcus aureus* with 0.39 mg mL^−1^ MIC value. The root hexane extract of *S. ceratophylla* manifested very significant antibacterial activity against *S. lutea* and *B. cereus* at a dose of 0.048 mg mL^−1^. It was effective against *Proteus* at 1.56 mg mL^−1^ MIC and *Candida* was more sensitive against root extract than aerial part hexane extract with 0.78 mg mL^−1^ MIC. When the dichloromethane extracts were evaluated it was determined that *S. lutea* affected from DCM aerial part extract at a dose of 0.097 mg mL^−1^ and affected from root extract at a concentration of 0.048 mg mL^−1^. MIC values were determined as 0.097 mg mL^−1^ both for two extract against *B. cereus*. Root extracts was more effective against *S. aureus* than aerial part extract with 0.097 mg mL^−1^. Two extracts of DCM affected *P. aeruginosa* at 1.56 mg mL^−1^ dose. Antifungal activity was observed at 3.12 mg mL^−1^ dose for two DCM extracts. The lowest MIC value was determined for methanol aerial part extract against *B. cereus* and *S. lutea* at a dose of 0.78 mg m^−1^. For root methanol extract *B. cereus* was the sensitive bacterium with 0.19 mg mL^−1^ MIC value. *Salmonella enteritidis*, which was resistant to hexane and DCM extracts, affected from aerial part methanol extract at 1.56 mg mL^−1^ concentration. Similarly, *Klebsiella pneumoniae* was sensitive to root methanol extract at a dose of 1.56 mg mL^−1^ while this bacterium resistant to hexane and DCM extracts. Except for *Yersinia enterocolitica* and *Salmonella typhimurium*, most of the bacteria showed MIC value ranging between 3.12-1.56 mg mL^−1^ concentrations against methanol extracts.

**Table 8:** Minimum inhibitory concentrations of *Salvia ceratophylla* extracts against pathogenic microorganisms

Strains	MIC values of Salvia ceratophylla extracts (mg mL^−1^)										Gentamicin (μg mL^−1^)
	Hexane		DCM		Methanol		Methanol/water		Aqueous		
	Aerial	Root	Aerial	Root	Aerial	Root	Aerial	Root	Aerial	Root	
*Escherichia coli* ATCC 25922	—	—	—	—	—	—	1.56	—	—	6.25	1.95
*Pseudomonas aeruginosa* ATCC 27853	—	1.56	1.56	1.56	1.56	1.56	1.56	1.56	—	—	<0.97
*Klebsiella pneumoniae* ATCC 70603	—	—	—	—	—	1.56	1.56	1.56	—	—	7.81
*Staphylococcus aureus* ATCC 43300	3.12	0.39	3.12	0.097	1.56	1.56	1.56	0.78	1.56	3.12	1.95
*Salmonella enteritidis* ATTC 13076	—	—	—	—	1.56	—	1.56	1.56	—	1.56	1.95
*Sarcina lutea* ATCC 9341	0.097	0.048	0.097	0.048	0.78	1.56	0.39	0.19	—		1.95
*Salmonella typhimurium* NRRLE 4463	—	—	—	—	—	—	1.56	1.56	—	—	1.95
*Yersinia enterocolitica* ATCC 1501	—	—	—	—	—	—	—	—	6.25	6.25	1.95
*Proteus mirabilis* ATCC 25933	3.12	1.56	3.12	3.12	3.12	1.56	1.56	1.56	3.12	3.12	1.95
*Bacillus cereus* ATTC 11778	0.19	0.048	0.097	0.097	0.78	0.19	0.39	0.097	—	—	1.95
*Citrobacter freundii* ATCC 8090	1.56	1.56	1.56	1.56	1.56	1.56	1.56	1.56	6.25	6.25	1.95
*Candida albicans* ATCC 26555	3.12	0.78	3.12	3.12	3.12	3.12	1.56	3.12	3.12	—	7.81

Methanol and water mixture aerial part extract of *S. ceratophylla* revealed MIC values between 3.12 to 1.56 mg mL^−1^ doses. Although MIC values for *S. lutea* and *B. cereus* were determined as 0.39 mg mL^−1^, this extract was effective against *S. typhimurium* at a dose of 1.56 mg mL^−1^ when compared previous three extracts. *Escherichia coli* only affected from aerial part extract at 1.56 mg mL^−1^ dose. The lowest MIC reported for root extract was 0.097 mg mL^−1^ for *B. cereus*. Infusion aerial part extract manifested antibacterial activity against *S. aureus* at a dose of 1.56 mg mL^−1^. Similarly, infusion root extract had antibacterial capacity against *S. enteritidis* (1.56 mg mL^−1^) only infusion extracts were effective against *Y. enterocolitica* with 6.25 mg mL^−1^ MIC value. The results showed that *S. ceratophylla* extracts had significant antibacterial activities against Gram positive bacteria (*B. cereus*, *S. lutea* and *S. aureus*) than Gram negative bacteria. Especially hexane and DCM root extracts revealed very good antibacterial activity against Gram positive bacteria at 0.048 mg mL^−1^ dose. The lowest MIC values were determined against *S. lutea* and *B. cereus*. The study showed that *Y. enterocolitica* and *E. coli* were the most resistant bacteria. *K. pneumoniae* affected from methanol-based extracts. Also extracts had antifungal capacity against *Candida albicans*. Hexane root extract showed the lowest antifungal activity at a dose of 0.78 mg mL^−1^.

Several *Salvia* species reported for their antimicrobial activity and pharmacological properties^37,38^ revealed that *Salvia* species contain caffeic acids, major group of phenolic acids, and derivatives. Caffeic acid plays a central role in the biochemistry of Lamiaceae and occurs predominantly in the dimer form as rosmarinic acid.^39^ The trimers and tetramers are also interesting from a therapeutic point of view as they have demonstrated various biological activities such as anti-oxidant, antimicrobial and anticancer.^40^ Chemical composition analyses showed that *S. ceratophylla* extracts tested in this assay included phenolic compounds such as rosmarinic acid and caffeic acid. In a study conducted by Matejczyk *et al.*,^41^ it was determined that caffeic acid revealed significant antimicrobial action against tested pathogens. Also, Li and Na salts of caffeic acid had an important activity, too. In that study also rosmarinic acid and its Li, Na and K salts were tested and better results were observed. Świsłocka^42^ reported that rosmarinic acid had bactericidal activity against *Staphylococcus epidermidis*, *Stenotrophomonas maltophilia*, and *Enterococcus faecalis.* Antimicrobial mechanisms of rosmarinic acid has not been explained clearly yet. But there were several studies about antibacterial mechanism of phenolic acids. The possible explanation for this situation could be as follows: the phenolic acids have pro-oxidative properties and they can alter the hydrophobicity and after the charging of the cell surface cellular cracking and formation can occur. The main mechanism of action of rosmarinic acid is its ability to damage the cell membrane.^43^ Significant antimicrobial activities of extracts determined in this study can be attributed to presence of rosmarinic and caffeic acid in *S. ceratophylla.*

### 3.5. Cytotoxicity effects

Plant-derived natural products have been considered as promising and potent chemotherapeutic agents for more than 40 years.^44^ In this study, the extracts of *S. ceratophylla* were evaluated against HepG2 (a human liver cancer cell line) and B164A5 (a skin melanoma cell line). The effects of the extracts on the viability of S17 cells, from non-tumoral origin, were also determined. Results are shown in Table 9.

**Table 9:** Cellular viability (%) of HepG2, B16 4A5 and S17 cell lines after application of the extracts of *Salvia ceratophylla* at the concentration of 100 μg mL^−1^^a^

Cell line	DMSO 0.5%	Aerial parts-MeOH	Aerial parts-aqueous	Roots-MeOH	Roots- aqueous
HepG2	101 ± 7^a^	75.3 ± 2.6^b^	89.4 ± 6.3^ab^	30.9 ± 2.5^c^	34.5 ± 1.7^c^
B16 4A5	88.2 ± 2.1^a^	90.4 ± 2.8^a^	57.3 ± 1.5^b^	95.1 ± 2.8^a^	91.1 ± 3.7^a^
S17	79.3 ± 4.9^b^	33.8 ± 2.7^c^	98.4 ± 1.0^a^	42.0 ± 1.2^c^	39.3 ± 3.4^c^
*SI – HepG2*	*0.79*	*0.45*	*1.10*	*1.36*	*1.14*
*SI – B16 4A5*	*0.90*	*0.37*	*1.72*	*0.44*	*0.43*

a Values represent the mean ± standard error of the mean (SEM) of six replicates (*n* = 6). HepG2 – human hepatocellular carcinoma cells; B16 4A5 – murine melanoma cells; S17 – murine bone marrow cells (normal cells); SI – selectivity index. In the same line, values marked by different letters are significantly different according to the Tukey HSD test (*P* < 0.05).

Root-MeOH and root-aqueous were the most toxic towards HepG2 cells (30.9 and 34.5% of cell viability), while extract aerial part-water was more active against B16 4A5 cells (57.3% of cell viability). Regarding the non-tumoral S17 cells, all samples showed significant toxicity, except extract aerial part-water that showed higher cell viability than the control (*P* < 0.05). Therefore, aerial part-aqueous although displaying moderate cytotoxic activity on B16 4A5 melanoma cells, exhibited the highest selectivity index for (SI = 1.72).

The observed results could be attributed to the presence phytochemicals present in the latter extract. For instance, this finding may be linked to the presence of gallic acid, which has been claimed to inhibit carcinogenesis and induces apoptosis in previous studies.^45–47^ Besides, the methanolic root extract contained luteolin, a flavonoid, also known to possess anti-cancer effect.^48–50^ However, as a future work, further assays should be conducted with the aim to isolate and identify the phytochemicals responsible for the observed cytotoxic properties and ensure if the toxicity towards cancerous cell lines is related to specific bioactive compounds.

### 3.6. PLS-DA based methods to discriminate between studied parts

**Fig. 3:**
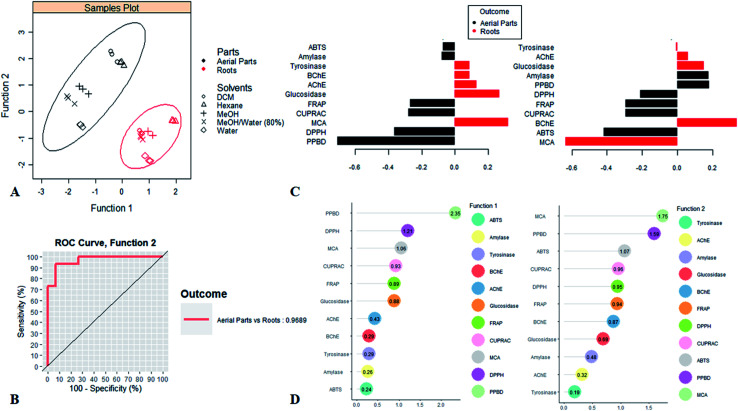
Partial least square discriminant analysis on biological activities of *Salvia ceratophylla.* (A) projection of samples into the subspace spanned by the first two function of PLS-DA. (B) The ROC (Receiver Operating Characteristic) curves assessing the prediction accuracy of a classification model. (C) Loadings plot showing the contribution of biological activities on the two function and the biological activities abundance among each parts. (D) discriminant biological activities identified by Variable Important in Projection (VIP).

The present study was focused upon two parts from *S. ceratophylla* including aerial part and roots and it was undertaken to assess the total antioxidant and selected five enzyme inhibitory activities of diverse extracts derived from said parts. For the purpose of evaluating the variation of antioxidant and enzyme inhibitory activities between the different studied parts, the supervised partial least squares discriminant analysis (PLS-DA) was applied to the data. PLS-DA is a multivariate regression analysis aiming at find the optimal linear combinations of variables being able accurately to discriminate the sample groups. In particular, latent function emanating from the linear combinations of variables summarize as much as possible the information and reduce the dimension of the original data. Thus, to perform the model, the factor “Parts” as used as class membership criteria and the results were reported in Fig. 3. By viewing Fig. 3A, we noted a clear discrimination between the two parts. The majority of aerial parts extracts were grouped on the left side of the first function while the roots extracts were aggregated on the positive and negative side of the first two function respectively. The model, had a great performance; in particular, incorporating the first two function, it was able to discriminate the both parts with an accuracy of 96.89% (Fig. 3B).

The loadings plot displayed the contribution of the biological activities on the first two function. Function 1 was positively related to MCA, glucosidase, AChE, BChE and tyrosinase and negatively bound to the other activities (PPBD, DPPH, FRAP, CUPRAC, Amylase and ABTS). While function 2 was positively determined by BChE, PPBD, amylase, glucosidase and AChE and negatively associated to MCA, ABTS, CUPRAC, FRAP, DPPH, and tyrosinase. On the other hand, this figure allowed to determine the biological activities characterizing each part. In general, antioxidant activities and anti-amylase recorded the highest value in aerial parts in contrast to roots that exhibited the best anti-cholinesterase, anti-glucosidase and anti-tyrosinase as well as metal chelating ability.

**Fig. 4:**
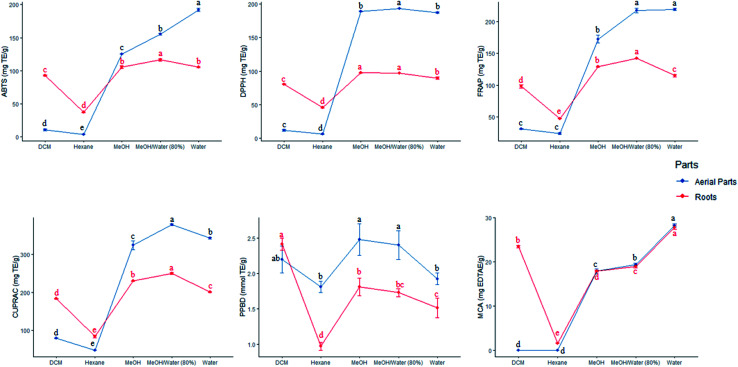
Effect of extraction solvents on the antioxidant activities of the tested extracts of each parts. TE: trolox equivalent; EDTAE: EDTA equivalent. (a–d) Column wise values with same superscripts of this type indicate no significant difference among extracts (*P* > 0.05).

Afterwards, the biological activities which mostly varied from one part to another were observed. In this regard, the VIP score of each bioactivity was calculated and reported in figure AC. On the basis of the value above 1, it emerged that four activities including PPBD, MCA, DPPH and ABTS, differed considerably across parts. Thus, aerial parts were characterized by an excellent total antioxidant capacity and ability to scavenging ABTS and DPPH radicals while roots were distinguished by a high ability to chelate Fe^2+^ ion (Fig. 4).

The results of the present study indicated high levels of bioactivities variability between the areal parts and roots of *S. ceratophylla.* The reason is that the concentration and type of secondary metabolites involve in the evaluated bioactivities, vary according to the plants parts. This outcome are in agreement with our previous work on the topic, which has reported that different parts of the same plant are characterized by different content of secondary metabolites.^51,52^ Further, this variability may be due to ordered expression of the genome such that specific enzymes or group of enzymes are activated for the biosynthesis of certain molecules at particular tissue or organ of plant, and not in another. For instance, Yosr *et al.*^53^ reported that the amount in leaves of phenolic compounds compared to the other plant organs may be due to the interaction between organs and multiple processes of synthesis or degradation and transport implied in the distribution of these phenolic compounds at the plant level.

### 3.7. Effect of extraction solvents on the antioxidant and enzyme inhibition activities of each parts

**Fig. 5:**
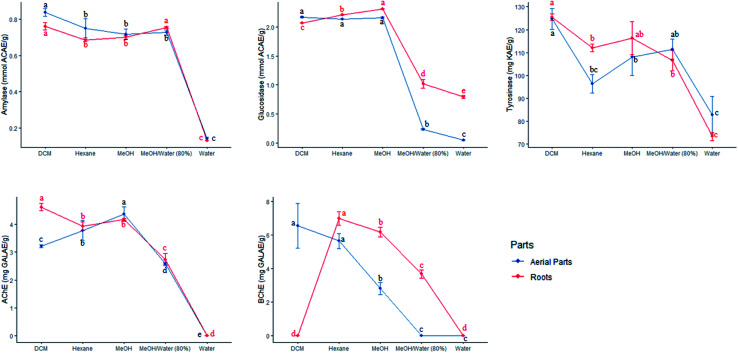
Effect of extraction solvents on the enzyme inhibitory activities of the tested extracts of each parts. GALAE: galatamine equivalent; KAE: kojic acid equivalent; ACAE: acarbose equivalent. (a–d) Column wise values with same superscripts of this type indicate no significant difference among extracts (*P* > 0.05).

Multiple solvents extraction condition was used with the purpose of achieving the best method to obtain a higher antioxidant and enzyme inhibitory activities of aerial parts and roots of *S. ceratophylla* (Fig. 3A and B). In general, a significant difference was observed between the extracts of each parts, for all biological activities. In aerial part, the extraction procedure using MeOH/water (80%) was highly efficient to scavenge DPPH radical and reduce Cu^2+^ ion. Similarly, as regards the roots, the same extracts exhibited highest ABTS scavenging capacity and Fe^3+^ and Cu^2+^ reducing power. Both methanol and MeOH/water (80%) extracts of roots and aerial parts scavenged DPPH radicals more effectively and presented highest total antioxidant capacity respectively. The extracts of aerial parts obtained using water possessed excellent ABTS and MCA activities while the water and MeOH/water (80%) showed a better reducing Fe^3+^ activity. Total antioxidant capacity of roots was ranged in order of DCM > MeOH > MeOH/water (80%) > water MeOH/water (80%) > hexane, whereas metal chelating activity increased as follows: water > DCM, MeOH/water (80%) > methanol > hexane. When it comes to enzyme inhibitory activities, hexane extract of aerial parts and roots had the highest anti-tyrosinase activity. In addition, the same extract exhibited strongest anti-BChE activity. However, in aerial parts, the activity of hexane extract was similar to that of DCM. For the second enzyme involved in the management of neurodegenerative disease, methanol (aerial parts) and DCM (roots) extractions showed the best anti-AChE activity. Regarding the anti-amylase assay, the strongest activity was shown by DCM for aerial parts and DCM and MeOH/water (80%) for roots. Furthermore, three extracts derived from aerial parts *i.e.*, DCM, hexane and MeOH showed the highest anti-glucosidase activity while regarding the roots the best activity was presented by MeOH (Fig. 5).

Historically, it is well known that extraction of secondary metabolites from plant matrix is impacted by multiple factors such as their chemical nature, the presence of interfering substances without forgetting the extraction solvent and technique used. In fact, the polarities of secondary metabolites in plants greatly vary and therefore, it is necessary to select an adequate solvent for efficient extraction in quantity and quality of the molecules of interest. As it is well known that secondary metabolites have diverse nature, concentration ranges and physicochemical properties. Accordingly, no single solvent able to recovery efficiently all of the classes of secondary metabolites from a plant matrix, simultaneously. This lends support our observations that the different solvent used, had showed each at least good result on all the evaluated biological activities. Moreover, outside the conventional extraction solvents, several researchers have employed combination of organic solvent-water for the extraction of secondary metabolites from plant. According to Cheng *et al.*,^54^ solvent mixtures allow to extract different molecules values, thanks to their differing efficacies in the penetration of plant matrixes and solubilization of the secondary metabolites. Much more, the presence of water enhance the permeability of cell membrane and therefore enables efficiently mass transfer by molecular diffusion as well as the extraction of the water soluble compounds.^54^

## 4. Conclusion

In the current work, all extracts of *S. ceratophylla* exhibited activity against amylase and glucosidase which are the key clinical enzymes related to diabetes, a disease affecting millions of people across the globe. A particular interest is the tyrosinase inhibitory activity displayed by the DCM root extract which can be qualified as a potent and promising activity. Thus, extract can further be examined for potential epidermal hyperpigmentation processes. Additionally, data amassed herein demonstrated that the hydro-methanolic aerial extract may act as a good antioxidant. From the antimicrobial analysis, it can be concluded that *S. ceratophylla* can be a potential source of bioactive compounds to combat *Bacillus cereus* infections. Methanolic root extract demonstrated a relatively low cytotoxicity. However, further toxicological studies should be conducted to ascertain its safety. The present study provides rationale for further *in vivo*/*ex vivo* pharmacological investigations.

## Author contributions

Sengul Uysal: conceptualization, data curation, formal analysis, writing – original draft. Gokhan Zengin: formal analysis, writing – original draft, supervision. Kouadio Ibrahime Sinan: methodology. Gunes Ak: methodology. Ramazan Ceylan: methodology. Mohamad Fawzi Mahomoodally: data curation, investigation, writing – original draft. Ahmet Uysal: methodology. Nabeelah Bibi Sadeer: data curation, investigation, writing – original draft. József Jekő: data curation, investigation. Zoltán Cziáky: data curation, investigation. Maria João Rodrigues: data curation, investigation. Evren Yıldıztugay: methodology. Fevzi Elbasan: methodology. Luisa Custodio: data curation, investigation.

## Conflicts of interest

There are no conflicts to declare.

## Supplementary Material
